# Potential Implications of Changing Photosynthetic End-Products of Phytoplankton Caused by Sea Ice Conditions in the Northern Chukchi Sea

**DOI:** 10.3389/fmicb.2019.02274

**Published:** 2019-10-02

**Authors:** Mi Sun Yun, Hyoung Min Joo, Jae Joong Kang, Jung Woo Park, Jae Hyung Lee, Sung-Ho Kang, Jun Sun, Sang H. Lee

**Affiliations:** ^1^College of Marine and Environmental Sciences, Tianjin University of Science and Technology, Tianjin, China; ^2^Tianjin Key Laboratory of Marine Resources and Chemistry, Tianjin University of Science and Technology, Tianjin, China; ^3^Department of Oceanography, Pusan National University, Busan, South Korea; ^4^Division of Polar Ocean Sciences, Korea Polar Research Institute, Incheon, South Korea; ^5^Graduate School of Fisheries Sciences, Hokkaido University, Hokkaido, Japan

**Keywords:** photosynthetic end-products, phytoplankton, carbon allocation, lipids, proteins, Arctic Ocean

## Abstract

The recent dramatic decline in sea ice conditions in the Arctic Ocean has led to the ecophysiological changes in the phytoplankton community. Little is currently known about how the physiological status of phytoplankton has changed under rapidly changing environmental conditions in the Arctic Ocean. Using the ^13^C isotope tracer technique, the carbon allocation of phytoplankton into different photosynthetic end-products was determined in the northern Chukchi Sea on the basis of two Arctic expeditions conducted in 2011 and 2012 to identify the physiological status of phytoplankton. Lipids were the predominant photosynthetic biochemical fraction (42.5%) in 2011, whereas carbon allocation to proteins was most dominant under ice-free conditions in 2012 (47.7%). Based on a comparison of the photosynthetic carbon allocation of phytoplankton according to sea ice conditions, we found that photosynthetic carbon allocation to different macromolecular pools was significantly different depending on the sea ice conditions and that the light conditions caused by different sea ice conditions could be an important reason for the differences in carbon allocation to photosynthetic end-products. Different dominant phytoplankton groups related to size classes also could cause changes in the photosynthetic carbon allocation of phytoplankton related mainly to the lipid synthesis. Our results showed that the physiological status of Arctic phytoplankton could be changed by producing different photosynthetic end-products under current environmental changes. This change in photosynthetic end-products of phytoplankton as a basic food source could be further linked to higher trophic levels in regards to their nutritional and energetic aspects, which could have potential consequences for Arctic marine ecosystems.

## Introduction

The dramatic decline of the sea ice extent in the Arctic Ocean has been pronounced over the past few decades (Stroeve et al., 2007; Comiso et al., 2008; Stroeve and Notz, 2015). According to continuous satellite observations, the downward trend of the September sea ice extent has accelerated over the past decade (Stroeve et al., 2007, 2012, 2014). In addition to the sea ice extent, sea ice thickness has changed, with high occupation of first-year ice, accounting for 60–70% of the Arctic Basin (Stroeve and Notz, 2018). The decline in sea ice cover and thickness has resulted in an increase in the amount of light penetrating the upper ocean (Arrigo and van Dijken, 2011). In addition, sea ice loss and combined rapid warming in the upper ocean has caused a delay in the fall freeze-up of sea ice and an increase in the melt season (Stroeve et al., 2012, 2014). Stroeve et al. (2014) found that the melt season in the Arctic lengthened at a rate of 5 days per decade from 1979 to 2013. These phenomena caused by sea ice changes have considerably affected the bloom timing (Kahru et al., 2011; Ardyna et al., 2017), community structure (Fujiwara et al., 2016) and productivity of phytoplankton in the Arctic Ocean (Arrigo and van Dijken, 2011, 2015; Kahru et al., 2016). Kahru et al. (2011) found that an earlier phytoplankton bloom is caused by sea ice loss. Ardyna et al. (2017) reported that the longer open water season in the Arctic has increased the incidence of autumn blooms. In addition, there is dominance of large-celled phytoplankton during the bloom period (Fujiwara et al., 2016). In ice-free Arctic waters, annual net primary production (NPP) based on satellite observations has also been largely increased (Arrigo and van Dijken, 2011, 2015; Kahru et al., 2016).

Compared to the many recent studies on the bloom timing, community structure, and productivity of phytoplankton, the scarcity of data on the physiological status of Arctic phytoplankton strongly limits our understanding of the impacts of changing environments on phytoplankton community characteristics in the Arctic Ocean. Very few studies have addressed about the effects of changing environmental conditions (e.g., irradiance or nutrient) on the photo-physiology or nutritional quality of phytoplankton (e.g., Van de Poll et al., 2005; Leu et al., 2006; La Rocca et al., 2015). Van de Poll et al. (2005) found that nutrient limitation in Antarctic diatom resulted in an increased value in the ratio between protective function and light-harvesting pigments, based on cultivation experiments. In addition, La Rocca et al. (2015) reported photo-physiological response of microalgae isolated from Antarctic Ocean under different light intensities. According to Leu et al. (2006), the fatty acid composition of phytoplankton in a high Arctic fjord was changed under high light intensities, especially during periods of stratification. Since the physiological status of primary producers can influence the nutritional conditions of higher trophic levels and lead to a significant change in marine ecosystems (Scott, 1980; Lindqvist and Lignell, 1997), it is important to monitor how the recent physiological status of phytoplankton has changed under the rapidly changing environmental conditions of the Arctic Ocean.

The relative proportions of carbon allocation among the different photosynthetic end-products (low-molecular-weight metabolites (LMWM), lipids, polysaccharides, proteins) of phytoplankton allow the assessment of the physiological state of an organism or population (Morris et al., 1974; Morris, 1981). According to Morris (1981) and Hitchcock (1983), the carbon allocation patterns of natural populations can reflect rapid adjustments to environmental conditions. Priscu and Priscu (1984) also suggested that photosynthetic carbon allocation patterns can be a useful index of the present cellular physiological state. For example, Yun et al. (2018) investigated carbon allocation patterns in the northern Chukchi Sea through stable carbon isotope (^13^C) analysis, showing lipid-rich and protein-poor characteristics in the northern Chukchi Sea during the summer of 2011. As a result, they revealed that the phytoplankton in the region were under nutrient-deficient conditions in some cases (Yun et al., 2018). The carbon allocation pattern in the same study area during 2012 was considerably different from that in 2011. Therefore, the different compositions of photosynthetic end-products were used for comparison of inter-annual variability in relation to environmental factors in the present study.

The objectives of the present study were to (1) describe the characteristics of the photosynthetic end-products of phytoplankton in the northern Chukchi Sea in 2011 and 2012 to identify the physiological status of phytoplankton under different sea ice conditions and (2) assess the influence of biotic and abiotic factors on the photosynthetic end-products of Arctic phytoplankton in the context of current environmental changes.

## Materials and Methods

### Study Area and Sampling Procedure

**FIGURE 1:**
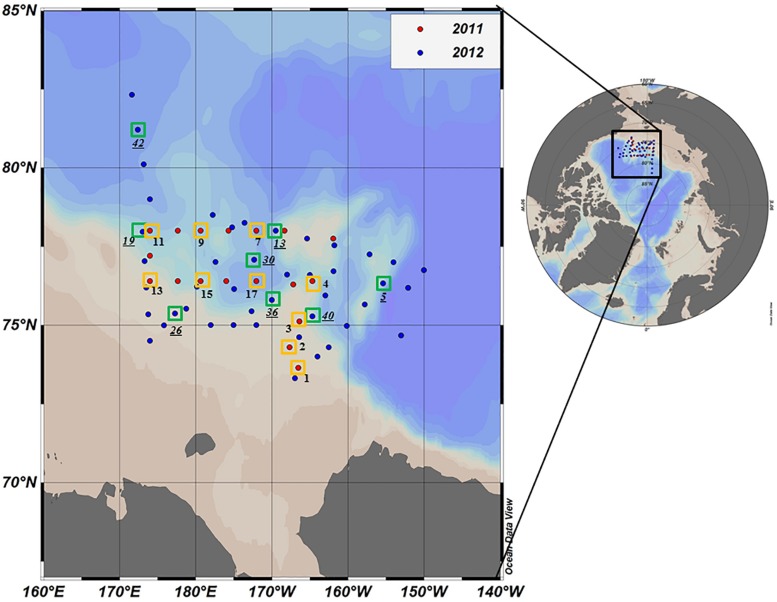
Location of the sampling stations in the northern Chukchi Sea during summer 2011 and 2012. Red and blue dots represent the stations visited in 2011 and 2012, respectively. The photosynthetic end-products were measured at the stations identified by yellow (2011) and green (2012) squares. Station ID in 2012 is underlined.

Oceanographic sampling was performed onboard the Korean Research Icebreaker *ARAON* in the northern Chukchi Sea from 30 July to 19 August 2011 and from 1 August to 10 September 2012 (Figure 1). Compared to 2011, a wider area including the Chukchi Cap and the western portion of the Northwind Ridge was covered during the longer study period in 2012 (Figure 1). A total of 18 and 50 stations were visited during the cruises in 2011 and 2012, respectively. For deck incubation, samples for determining the photosynthetic end-products of phytoplankton were collected from 10 and 8 selected morning stations in 2011 and 2012, respectively (Figure 1 and Table 1).

**TABLE 1:** Environmental variables at stations where photosynthetic end-product measurements were conducted in the northern Chukchi Sea during summer 2011 and 2012.

**Year**	**Station**		**Environmental variable**									
		
		**Date (dd/mm/yy)**	**T_eu_ (°C)**	**S_eu_**	**△σ_t_ (kg m^–3^)**	**I_c_ (%)**	**Z_eu_ (m)**	**Z_m_ (m)**	**NO_3_ + NO_2_ (μM)**	**NH_4_ (μM)**	**SiO_2_ (μM)**	**PO_4_ (μM)**
2011	1	02/08/2011	−0.4	31.1	2.3	50	38	5	1.74	0.56	8.96	0.81
	2	03/08/2011	−0.9	29.7	4.7	<30	54	11	1.32	0.64	4.14	0.85
	3	04/08/2011	−1.2	30.4	5.0	30–40	92	14	3.94	0.54	11.38	0.95
	4	05/08/2011	−0.7	30.2	5.0	70–80	100	12	3.14	0.44	8.57	0.94
	7	08/08/2011	−1.1	30.9	3.8	<10	87	10	3.46	0.29	9.33	1.01
	9	10/08/2011	−1.1	30.9	3.3	90	79	15	4.24	0.68	9.72	1.01
	11	11/08/2011	−1.2	30.7	3.0	80	60	16	2.24	0.60	6.38	0.90
	13	13/08/2011	−1.4	30.5	2.6	>90	57	9	1.62	0.60	6.03	0.95
	15	14/08/2011	−1.3	30.5	3.1	80	81	27	1.56	0.57	6.42	0.93
	17	15/08/2011	−0.7	30.2	4.7	<10	98	13	1.94	0.46	6.59	0.88
2012	5	01/09/2012	0.0	28.9	5.1	0	68	20	1.02	0.83	4.74	0.73
	13	09/08/2012	−0.8	29.5	3.7	60	65	23	1.45	0.71	5.50	0.73
	19	17/08/2012	−1.1	29.8	3.7	5	43	17	1.95	0.45	6.34	0.73
	26	21/08/2012	−1.0	30.3	4.6	10	51	15	3.90	0.55	12.54	0.98
	30	25/08/2012	−0.6	29.5	4.7	5	65	17	2.74	1.05	6.33	0.85
	36	29/08/2012	−0.4	29.3	5.5	0	68	17	1.57	0.67	5.61	0.80
	40	05/08/2012	−0.6	29.4	5.5	5	76	4	2.62	0.98	5.84	0.66
	42	16/08/2012	−1.4	28.5	5.2	60	41	3	0.71	0.00	15.38	0.60

*T_*eu*_, water temperature averaged over the depth of the euphotic zone (Z_*eu*_); S_*eu*_, salinity averaged over Z_*eu*_; Δσ_*t*_, stratification index; I_*c*_, percent areal ice cover; PAR, photosynthetically available radiation; Z_*m*_, surface mixed layer depth; NO_3_ + NO_2_, nitrate plus nitrite concentration averaged over Z_*eu*_; SiO_2_, silicate concentration averaged over Z_*eu*_; PO_4_, phosphate concentration averaged over Z_*eu*_.*

**FIGURE 2:**
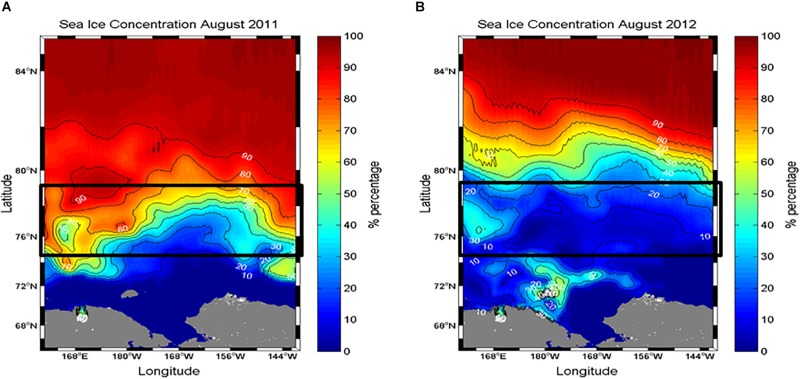
Comparison of sea ice conditions in the northern Chukchi Sea during **(A)** August 2011 and **(B)** 2012. Sea ice concentration data were derived from the Special Sensor Microwave/Imager (SSM/I) using the NASA Earth Observing System (EOS) Aqua satellite. Data were obtained from the National Snow and Ice Data Center (http://nsidc.org).

Sea ice coverage (I_c_) at each experimental station was estimated from shipboard visual observations performed by ice-navigators (Table 1). In addition, sea ice concentration data during two cruise periods were obtained from the National Snow and Ice Data Center (NSIDC) (Figure 2).

A vertical profile of irradiance (PAR: photosynthetically active radiation, 400 to 700 nm) was obtained with an underwater PAR sensor (LI-COR underwater 4π light sensor) mounted on the CTD/rosette sampler, and was then used to determine the depth of the euphotic zone (Z_eu_, as the depth receiving 1% of the surface PAR) as in Lee et al. (2007) and Yun et al. (2014).

At each station, water samples were collected with a rosette sampler equipped with 20 L Niskin-type bottles, a Sea-Bird 911 plus conductivity, temperature, depth (CTD) probe for temperature and salinity measurements, and a chlorophyll fluorometer (Seapoint). Water samples were collected at three (100, 30, and 1% penetration of surface irradiance) or six optical depths (100, 50, 30, 12, 5, and 1% penetration of surface irradiance).

### Nutrients, Chlorophyll a, and Particulate Organic Carbon and Nitrogen

Nutrient and total chlorophyll a (chl a) concentrations were determined at six optical depths (100, 50, 30, 12, 5, and 1%). Samples for size-fractionated chl a concentration and particulate organic carbon and nitrogen (POC and PON) determinations were only obtained at three optical depths (100, 30, and 1%).

Nitrate plus nitrite (NO_3_ + NO_2_), ammonium (NH_4_), phosphate (PO_4_), and silicate (SiO_2_) concentrations were measured onboard immediately after collection using an automated nutrient analyzer (SEAL, QuAAtro, United Kingdom) following the QuAAtro multitest methods.

Samples for the measurement of total chl a concentrations (referred to as total phytoplankton biomass) were filtered through Whatman GF/F filters (24 mm). The size-fractionated chl a concentration was determined by sequentially passing samples through 20- and 5-μm Nucleopore filters (47 mm) and 0.7-μm Whatman GF/F filters (47 mm), and the different fractions are referred to as the biomass of large, medium, and small phytoplankton cells, respectively. Chl a concentrations were measured using a Trilogy fluorometer (Turner Designs, United States), which had been calibrated with commercially purified chl a preparations following a 24 h extraction in 90% acetone at 4°C in the dark without grinding (Parsons et al., 1984).

Samples for the determination of POC and PON concentrations were filtered through precombusted Whatman GF/F filters and then immediately frozen at −80°C. A 300 ml or 400 ml sample was filtered depending on the quantity of particulate matter in the sample. The total contents of POC and PON after treatment with HCl fumes overnight to remove inorganic carbon (Hama et al., 1983) were determined in a Thermo Finnigan Delta +XL mass spectrometer at the stable isotope laboratory of the University of Alaska, Fairbanks.

### Photosynthetic End-Products of Phytoplankton

The stable carbon isotope (^13^C) was utilized to trace photosynthetic carbon allocation into different macromolecular classes as photosynthetic end-products of phytoplankton (Slawyk et al., 1977; Lee et al., 2009). At three optical depths (100, 30, and 1%) within euphotic zone, water samples were taken in polycarbonate incubation bottles (8.8 L), which were covered with screens, and NaH^13^CO_3_ solution (99%) was immediately added to the polycarbonate incubation bottles at a final concentration of ∼0.2 mM (Hama et al., 1983). Then, the water samples were incubated on deck for approximately 4–7 h. The changes in cell status during incubation were not monitored. After incubation, all the samples were gently filtered through pre-combusted (450°C) Whatman GF/F filters (diameter = 47 mm), and the filtered samples were stored at −80°C until further analysis. The differential extraction of incorporated carbon into the four main macromolecular classes (LMWM, lipids, polysaccharides, and proteins) was based on the methods of Li et al. (1980, 2009). We followed the same analytical procedure as Lee et al. (2009) and Yun et al. (2018) to measure the different macromolecular classes to consistently compare the carbon allocation pattern determined in the two studies. Briefly, a solution of chloroform-methanol (2:1 v/v) was used to extract LMWM and lipids from phytoplankton on the filters. For the determination of polysaccharides and proteins, a 5% trichloroacetic acid (TCA) solution was added. The atom% of ^13^C in each extracted macromolecular class was analyzed with a Thermo Finnigan Delta + XL mass spectrometer at the Alaska Stable Isotope Laboratory (see Yun et al., 2018 for details).

### Phytoplankton Abundance and Taxonomic Composition

Samples for the taxonomic enumeration and identification of phytoplankton were obtained from different water depths for the assessment of photosynthetic carbon allocation (100, 30, and 1% penetration of surface irradiance, PAR). Water samples (125 mL) were taken from the Niskin bottles and immediately fixed with glutaraldehyde (final concentration of 1%). Sample volumes of 50–100 mL were filtered, and the filters were mounted using HPMA (2-hydroxypropyl methacrylate) on board (Crumpton, 1987). HPMA slides are generally used for obtaining quantitative estimates of pico- and nano-size microalgae (Joo et al., 2012; Lee et al., 2015). In the laboratory, phytoplankton cells were identified and counted with a combination of light and epifluorescence microscopy (BX51, Olympus Inc., Tokyo, Japan) following the procedure of Joo et al. (2012). SEM (scanning electron microscope; JSM-5600LV, JEOL) also was used for species that could not be identified with light microscopy. The main taxonomic reference used to identify the phytoplankton was Tomas (1997).

The Shannon-Wiener diversity index was used to obtain phytoplankton diversity. To identify the effects of different phytoplankton groups on carbon allocation patterns into photosynthetic end-products, the biovolume estimates of each phytoplankton were based on cell dimensions measured by light microscopy and SEM using appropriate geometric shapes according to Sun and Liu (2003). The each phytoplankton were grouped into three main taxonomic groups based on size classes, defined as the Diatoms (Bacillariophyceae), Flagellates (Dinophyceae, Cryptophyceae, Chrysophyceae, Dictyochophyceae, Prasinophyceae, and Prymnesiophyceae), and unidentified phytoplankton (unidentified nano- and pico-sized species).

### Calculations and Statistical Analyses

Water temperature and salinity were averaged over the euphotic zone (referred to as T_eu_ and S_eu_, respectively). The stratification index of the upper water column (Δσ_t_) (in kg m^–3^) was determined as the difference in σ_t_ values between 80 and 5 m, as in Tremblay et al. (2009). The surface mixed layer (Z_m_) was defined as the depth at which the density (σ_t_) gradient was 0.05 kgm^–3^ higher than the surface density, as in Coupel et al. (2015). Major inorganic nutrient concentrations were averaged over Z_eu_. Chl a concentrations were integrated over the euphotic zone using trapezoidal integration (Knap et al., 1996).

For each variable, one-way analysis of variance (ANOVA) was performed to assess significant differences in the environmental and biological variables of the two sampling years (i.e., 2011 and 2012). Principle component analysis (PCA) was conducted to assess the interactions of the variability of various environmental factors and biovolume in each group (diatoms, flagellates, and unidentified phytoplankton) and relate them to the changes in photosynthetic carbon allocation. Two ordination diagrams were produced to visualize interactions between photosynthetic biochemical fractions in relation to (1) three taxonomic groups and (2) environmental variables. These statistical tests were carried out using SPSS 12.0 software for Windows.

## Results

### Sea Ice Conditions in 2011 and 2012

The different sea ice concentrations obtained from satellite data were clearly detected between the 2 years (Figure 2). The study area in August 2011 was covered by sea ice with high concentrations (Figure 2A). In comparison, the satellite observations showed that the sea ice in the same period of 2012 consistently retreated in a northward direction, so the study area was almost ice-free with a concentration of less than 10% (Figure 2B). These observations are consistent with the areal coverage of sea ice at each experimental station (Table 1).

### Vertical Structures of Temperature and Salinity

**FIGURE 3:**
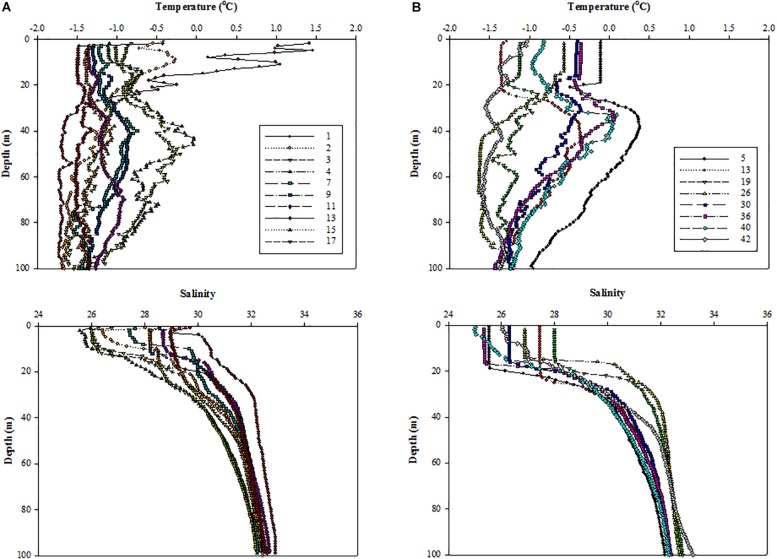
Vertical structures of temperature (upper panel) and salinity (lower panel) at all the experimental stations in the northern Chukchi Sea in **(A)** 2011 and **(B)** 2012.

The vertical profiles of temperature and salinity showed that all surface waters were at freezing temperatures except at station 1 (Figures 3A,B). Compared to 2011, the salinity in the upper ocean was significantly lower under ice-free conditions in 2012 (Figure 3B). In 2012, all the surface waters presented well-mixed conditions within the upper 20 m except at stations 40 and 42 (Figure 3B).

### Physical and Chemical Environments Within the Euphotic Zone

The environmental variables measured in the northern Chukchi Sea during the summer season showed that the mean water temperature in the euphotic zone (T_eu_) ranged from −1.4 to −0.4°C, and salinity (S_eu_) ranged from 29.7 to 31.1 psu among the stations in 2011 (Table 1). In 2012, T_eu_ and S_eu_ averaged over the euphotic zone ranged from 0.0 to −1.4°C and 28.5 to 30.3 psu in the study area, respectively (Table 1). The stratification index (Δσ_t_) in the study area ranged from 2.3 to 5.0 with a mean of 3.8 ± 1.0 kg m^–3^ in 2011 and from 3.7 to 5.5 with a mean of 4.8 ± 0.7 kg m^–3^ in 2012. The mean Z_eu_ was 75 ± 21 m, ranging from 38 to 100 m among the stations for the measurement of photosynthetic end-products in 2011. In 2012, the mean Z_eu_ was 60 ± 13 m. The surface mixed layer in 2011 varied between 5 and 27 with a mean of 13 ± 6 m among the productivity stations. In 2012, the surface mixed layer ranged from 3 to 23 with a mean of 15 ± 7 m among the stations.

The water column ambient nitrate + nitrite concentrations averaged over the euphotic zone were slightly different (Table 1). In 2011, the nitrate + nitrite concentrations averaged over the euphotic zone ranged from 1.32 to 4.24 μM, with a mean of 2.52 ± 1.08 μM. In comparison, the mean nitrate + nitrite concentration in 2012 was 2.00 ± 1.05 μM, ranging from 0.71 to 3.90 μM. The mean ammonium concentrations averaged within the euphotic zone were not considerably different among the stations in 2011 and 2012 (0.54 ± 0.11 and 0.66 ± 0.33 μM, respectively). In 2011, the silicate concentrations ranged from 4.14 to 11.38 μM, whereas the phosphate concentrations ranged from 0.81 to 1.01 μM. In comparison, the silicate and phosphate concentrations in 2012 ranged from 4.74 to 15.38 μM and from 0.60 to 0.98 μM, respectively.

### Phytoplankton Biomass

In 2011, the total (>0.7 μm) phytoplankton biomass (B_T_) was highest at station 2 (120.6 mg m^–2^) followed by station 1, with a value of 86.3 mg m^–2^. Apart from stations 1 and 2, the other stations in the region showed evenly low phytoplankton biomass (Table 2). At the 8 stations where photosynthetic end-products were measured in 2012, B_T_ ranged from 6.4 mg m^–2^ to 60.9 mg m^–2^ and was fairly low within the euphotic zone at most stations (<20 mg m^–2^), except for station 26 (Table 2). The station-averaged B_T_ values were 37.5 ± 35.9 mg m^–2^ and 17.8 ± 17.8 mg m^–2^ in 2011 and 2012, respectively.

**TABLE 2:** Phytoplankton biomass in the northern Chukchi Sea in 2011 and 2012.

**Year**	**Station**	**B_T_ (mg chl a m^–2^)**	**Different size fractions (mg chl a m^–2^)**		
			**B_L_**	**B_M_**	**B_S_**
2011	1	86.3	58.3	3.6	4.6
	2	120.6	136.7	4.7	17.8
	3	16.2	2.8	1.5	3.3
	4	15.0	0.6	0.6	2.4
	7	21.9	0.5	1.2	4.4
	9	21.0	0.5	1.1	4.6
	11	24.4	0.5	1.7	7.5
	13	22.2	5.2	3.0	9.3
	15	24.8	1.2	3.5	8.1
	17	22.2	0.4	0.7	3.5
2012	5	14.8	6.8	2.2	7.0
	13	17.0	2.4	1.5	10.4
	19	10.3	3.6	1.8	5.1
	26	60.9	38.7	7.7	5.1
	30	6.4	0.6	0.7	4.5
	36	14.2	1.8	2.7	9.2
	40	11.4	0.6	1.1	8.0
	42	7.5	0.4	0.8	5.3

*B_*T*_, total phytoplankton biomass (>0.7 μm); B_*L*_, biomass of large phytoplankton (>20 μm); B_*M*_, biomass of medium phytoplankton (5–20 μm); B_*S*_, biomass of small phytoplankton (0.7-5 μm). Total and size-fractionated chl a data were integrated over the euphotic zone.*

Based on the concentrations of chl a fractionated by size at each station, the large phytoplankton biomass (B_L_, > 20 μm) ranged from 0.4 to 136.7 mg chl a m^–2^ in 2011 (Table 2). The medium (B_M_, 5–20 μm) and small phytoplankton biomasses (B_S_, 0.7–5 μm) were 0.7–4.7 mg chl a m^–2^ and 2.4–17.8 mg chl a m^–2^, respectively. In comparison, the size-fractionated phytoplankton biomasses in 2012 were 0.4–38.7 mg chl a m^–2^, 0.7–7.7 mg chl a m^–2^ and 4.5–10.4 mg chl a m^–2^ for the large, medium and small cells, respectively (Table 2).

### Phytoplankton Composition, Abundance, and Biovolume

The number of phytoplankton species ranged from 1 to 13 species, excluding unidentified nano-phytoplankton (2–20 μm) and pico-phytoplankton (0.7–2 μm), at the three optical depths in the euphotic zone in 2011. The diversity index ranged from 0.05 to 1.53 at the three optical depths. The total phytoplankton cell abundance at the three optical depths at all the stations ranged from 1.7 × 10^5^ to 1.8 × 10^7^ cells L^–1^, with a mean of 1.5 × 10^6^ ± 3.2 × 10^6^ cells L^–1^ (Supplementary Table S1). In 2012, the phytoplankton species number ranged from 1 to 24 species, excluding unidentified nano-phytoplankton and pico-phytoplankton. The phytoplankton diversity varied from 0.37 to 1.51 among stations in 2012. Excluding the 100% optical depth at station 26, for which no data were available, the total phytoplankton cell abundance at the three optical depths ranged from 3.5 × 10^5^ to 5.4 × 10^6^ cells L^–1^, with a mean of 1.3 × 10^6^ ± 1.3 × 10^6^ cells L^–1^) (Supplementary Table S2).

**FIGURE 4:**
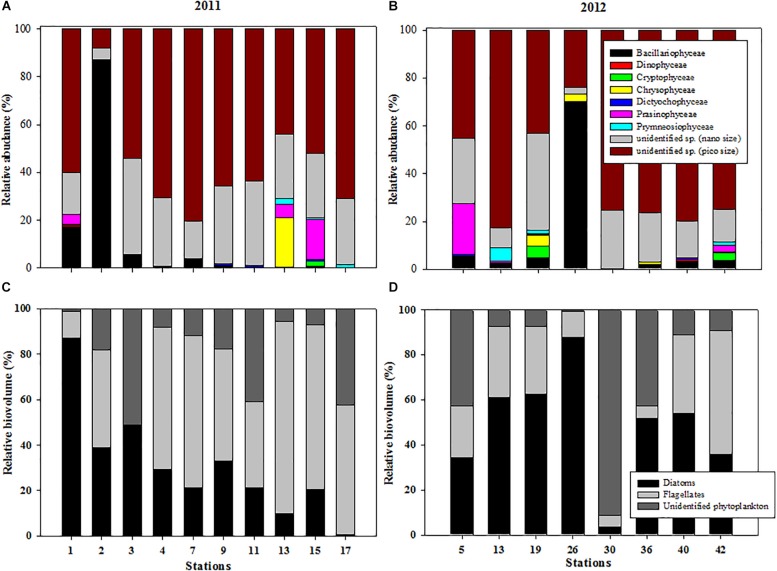
Variations in the relative abundance of taxonomic groups at each station during **(A)** 2011 and **(B)** 2012 and relative biovolumes of three phytoplankton groups (diatoms, flagellates, and unidentified nano- and pico-sized phytoplankton) at each station in the northern Chukchi Sea during **(C)** 2011 and **(D)** 2012.

Regarding the variations in the relative abundance of different taxonomic groups at each station, nano- and pico-sized phytoplankton was dominant at most stations in 2011, and together they accounted for 70.8 to 99.1% of the relative abundance of total phytoplankton (Figure 4A). Exceptionally, the diatom contributed up to 86.9% of the total phytoplankton abundance at station 2 (Figure 4A). In 2012, we observed that the phytoplankton community was also dominated by nano- and pico-phytoplankton, except at station 26. The nano- and pico-phytoplankton contributed approximately 90% of the total phytoplankton abundance, with the exception of station 26 (Figure 4B).

The proportions of the total biovolume represented by the three phytoplankton groups showed that the diatoms contributed approximately 31.1 ± 24.0%, whereas the flagellates contributed 48.6 ± 26.5% of the three phytoplankton groups in 2011 (Figure 4C). Unidentified nano- and pico-sized phytoplankton contributed 20.4 ± 17.8%. Across the study area, the phytoplankton community was characterized by prevalence of the diatoms in 2012 (Figure 4D). The highest biovolume was observed in the diatoms, accounting for 49.0 ± 24.8%, followed by unidentified nano- and pico-phytoplankton (26.5 ± 30.8%) and flagellates (24.5 ± 17.0%) (Figure 4D).

### Photosynthetic Carbon Allocation Into Different Macromolecular Classes

**FIGURE 5:**
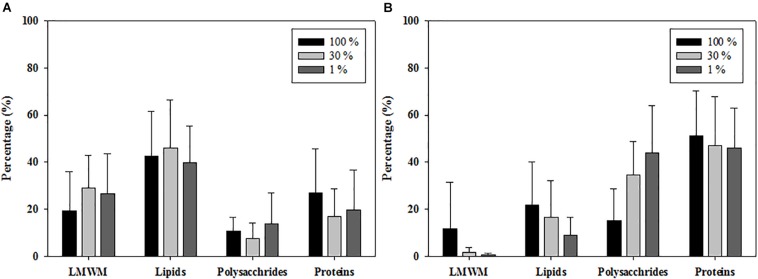
Photosynthetic carbon allocations of phytoplankton at 100, 30, and 1% light depths averaged from all the experimental stations in the northern Chukchi Sea in **(A)** 2011 and **(B)** 2012.

The carbon allocation pattern was distinctly different between the 2 years (Figure 5). In 2011, the contributions of LMWM production at the three optical depths ranged from 19.6 to 29.3% (mean ± S.D. = 26.1 ± 15.5%), and the contributions of lipids ranged from 39.8 to 46.0% (mean ± S.D. = 42.5 ± 17.7%) (Figure 5A). Polysaccharide allocation ranged from 7.7 to 13.8% (mean ± S.D. = 11.0 ± 10.0%), whereas the contributions of proteins ranged from 17.1 to 27.1%, with a mean of 20.4 15.5% (Figure 5A). In comparison, the LMWM allocation at the three optical depths in 2012 ranged from 0.6 to 11.8% (mean ± S.D. = 3.9 ± 10.6%) (Figure 5B). The carbon allocation into lipids and polysaccharides ranged from 9.2 to 21.8% (mean ± S.D. = 14.9 ± 13.9%) and from 15.1 to 44.1% (mean ± S.D. = 33.5 ± 19.9%), respectively. The contributions of protein allocation were highest, with a mean of 47.7%, ranging from 46.0 to 51.3% in 2012 (Figure 5B).

### Relationships Among Photosynthetic Biochemical Fractions

**FIGURE 6:**
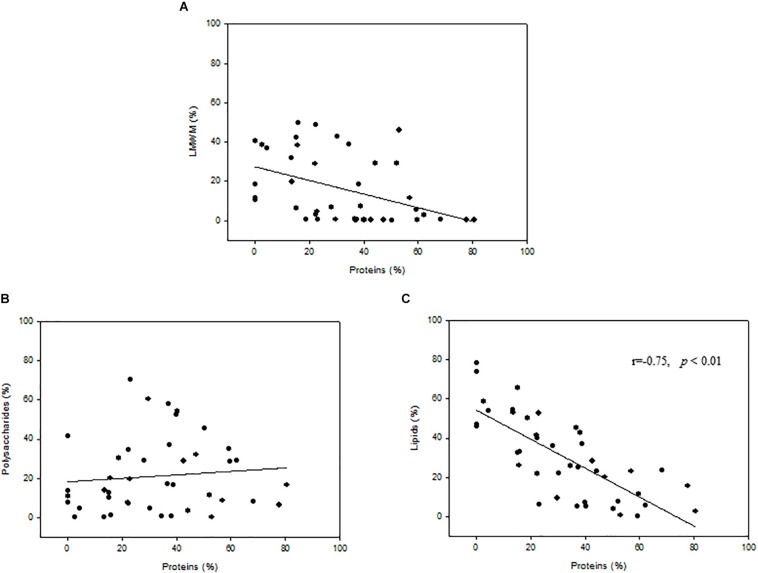
Relationships between proteins(%) and percent carbon incorporation into **(A)** low-molecular-weight metabolites (LMWM), **(B)** polysaccharides, and **(C)** lipids.

The relationships between protein fractions and the percent carbon allocation into LMWM, lipids, and polysaccharides indicated that no significant correlations were found for the LMWM and protein fractions or the polysaccharide and protein fractions (Figures 6A,B). However, correlation analysis revealed statistically significant relationships between the protein and lipid fraction (*r* = −0.75, *p* < 0.01, *n* = 42) (Figure 6C). A negative correlation also existed between the LMWM and polysaccharide fractions (*r* = −0.66, *p* < 0.01, *n* = 42) (data not shown).

### Statistical Analysis

The statistical results revealed significant yearly differences for some of the environmental and biological variables in the study area (Table 3). Due to largely different sea ice conditions, the salinity averaged over the euphotic zone was significantly different in the region (*p* < 0.001). The stratification index also exhibited significant variability in the two sampling years (*p* < 0.05). However, T_eu_ was similar in the two sampling years. The surface mixed layer depth and euphotic depth were also not significantly different (Table 3). The ambient nutrient concentrations averaged over the euphotic zone were not considerably different between the two sampling years, although the phosphate concentration was significantly different between the two sampling periods (*p* < 0.01) (Table 3). During this study, there were no significant inter-annual differences in the ratios of (NO_3_ + NO_2_ + NH_4_):PO_4_ and (NO_3_ + NO_2_ + NH_4_):SiO_2_ (Table 3). For the biological variables, total and size-fractionated phytoplankton biomasses did not show any significant inter-annual differences, even though the mean total phytoplankton biomass was considerably different (Table 3). In addition, there was no significant difference in the total abundance of phytoplankton between the 2 years. However, the relative biovolume of flagellates was significantly greater in 2011 than in 2012 (*p* < 0.05) (Table 3).

**TABLE 3:** Summary of one-way analysis of variance (ANOVA) for environmental and biological variables of the Chukchi Sea in 2011 and 2012.

**Variables**	**2011**		**2012**
**Environmental variables**			
T_eu_ (°C)	−1.0	ns	−0.7
S_eu_	30.5	>^∗∗∗^	29.4
Δσ_t_ (kg m^–3^)	3.8	<^∗^	4.8
Ic (%)	75.0	>^∗∗∗^	18
Z_eu_ (m)	74.6	ns	59.6
Z_m_ (m)	13	ns	15
NO_2_ + NO_3_ (μmol l^–1^)	2.52	ns	2.00
NH_4_ (μmol l^–1^)	0.54	ns	0.66
SiO_2_ (μmol l^–1^)	7.75	ns	7.79
PO_4_ (μmol l^–1^)	0.92	>^∗∗^	0.76
(NO_2_ + NO_3_ + NH4)_:_ PO_4_ (mol:mol)	2.54	ns	2.74
(NO_2_ + NO_3_ + NH4)_:_ SiO_2_ (mol:mol)	0.31	ns	0.36
**Biological variables**			
B_T_	37.5	ns	17.8
B_L_	20.7	ns	6.9
B_M_	2.2	ns	2.3
B_S_	6.6	ns	6.8
Total abundance (10^6^ cells l^–1^)	1.5	ns	1.4
Diatoms (%)	31.1	ns	49.0
Flagellates (%)	48.6	>^∗^	24.5
Unidentified phytoplankton (%)	20.4	ns	26.5

*The mean values and their significant differences (> or <) between 2011 and 2012 are given. Total abundance: total abundance of phytoplankton averaged over the euphotic zone; Diatoms: relative biovolume of diatoms averaged over the euphotic zone; Flagellates: relative biovolume of flagellates averaged over the euphotic zone; Unidentified phytoplankton: relative biovolume of unidentified nano- and pico-phytoplankton averaged over the euphotic zone. Other abbreviations are defined in Tables 1, 2. ^∗^*p* < 0.05, ^∗∗^*p* < 0.01, ^∗∗∗^*p* < 0.001, ns: not significant. *n* = 18.*

**FIGURE 7:**
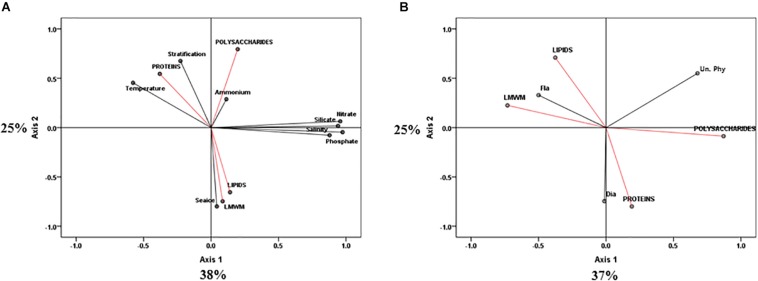
Principle component analysis (PCA) ordination plots of axes I and II showing photosynthetic biochemical fractions (red arrows) in relation to **(A)** environmental (black arrows) and **(B)** taxonomic group (black arrows) variables in the northern Chukchi Sea during 2011 and 2012. Dia, diatoms biovolume; Fla, flagellates biovolume; Un. Phy, unidentified nano plus pico biovolume.

Constrained principal component analysis (PCA) with environmental variables resulted in first (PC1) and second (PC2) principal components that accounted for 38 and 25% of the total variance, respectively (Figure 7A). PC1 clearly explained the changes in environmental factors such as temperature, salinity and nutrient concentrations (nitrate, phosphate, and silicate). PC2 explained changes in sea ice conditions and stratification. Most of the variations of the photosynthetic end-products were also revealed by PC2 (Figure 7A). Both lipids and LMWM were negatively related to PC2, whereas proteins and polysaccharides were positively related to PC2. Lipids and LMWM were strongly related to sea ice conditions (Figure 7A). In terms of the effects of different phytoplankton groups on photosynthetic biochemical fractions, PC1 and PC2 accounted for 37 and 25% of the total variance, respectively (Figure 7B). We found that flagellate abundance was negatively related to PC1, whereas diatom abundance was strongly negatively related to PC2 (Figure 7B). The results showed that there were close relationships between lipids, LMWM and flagellates. Proteins were strongly related to the change in diatom proportions.

## Discussion

### Interannual Variability of Environmental Conditions

Despite similar sampling coverage during the two study years, some differences in environmental conditions were observed. Notably, sea ice coverage in the study area was different in 2011 and 2012 (Figure 2). Considerable sea ice cover existed at most of the experimental stations in 2011, whereas the study area in 2012 showed almost ice-free conditions, although the northernmost station had approximately 60% sea ice cover (Table 1 and Figure 2). Due to distinct differences in sea ice coverage, salinity differed significantly between the 2 years (*p* < 0.001) (Table 3). Under ice-free conditions, water stratification was also enhanced in 2012 compared to 2011 (Tables 1, 3).

Generally, stronger stratification prevents the supply of nutrients from deep water, while the establishment of a thick freshwater layer deepens the nitracline (Coupel et al., 2012). As a result, low phytoplankton biomass and primary productivity can be expected under strong stratification (Yun et al., 2014, 2016). However, there were no significant differences in dissolved inorganic nitrogen concentrations between the 2 years, even though stronger stratification was observed in 2012 (Table 3). Average phytoplankton biomass (chl a) also were not significantly different, even though they had a slight variation depending on stations (Table 3). Based on comparison C:chl a between the 2 years, we found that C:chl a in 2012 was two-times higher than that in 2011. This could be due in part to the elevated POC concentration in 2012, since the chl a were similar between the 2 years. Thus, this means that phytoplankton in 2012 produced more carbon per unit chl a responding to more abundant light conditions under the ice-free environments.

Interestingly, the average molar ratios of (NO_3_ + NO_2_ + NH_4_):PO_4_ and (NO_3_ + NO_2_ + NH_4_):SiO_2_ over the euphotic zone were similar between the 2 years, at 2.54 ± 0.73 and 0.31 ± 0.07 for 2011 and 2.74 ± 1.10 and 0.36 ± 0.20 for 2012, respectively. These ratios were considerably lower than the Redfield-Brzezinski values (16 and 1, respectively; Redfield et al., 1963; Brzezinski, 1985; Conley and Malone, 1992). Moreover, the average molar ratios of (NO_3_ + NO_2_ + NH_4_):PO_4_ at 1% optical depths were 6.32 ± 1.44 in 2011 and 5.22 ± 1.80 in 2012, although the average molar ratios of (NO_3_ + NO_2_ + NH_4_):PO_4_ at 1% optical depths were ca. 4.6 and 2.7 times higher than at the sea surface (100% optical depths) in 2011 and 2012, respectively. Yun et al. (2015a) also reported the very low nutrient condition in the upper layers of the western Arctic Ocean. Therefore, this finding suggests possible nitrogen shortage conditions throughout the euphotic zones in the study region in 2011 as well as 2012, when it was under ice-free and more stratified conditions.

### Factors Affecting Different Patterns of Photosynthetic Carbon Allocation Between 2011 and 2012

In the northern Chukchi Sea, the photosynthetic end-products of phytoplankton were different between the 2 years. Lipids were the predominant photosynthetic biochemical fraction (42.5%) in 2011, whereas carbon allocation into proteins was most dominant in 2012 (47.7%) (Figures 5A,B). Generally, the carbon allocation patterns in photosynthetic end-products of the phytoplankton community can be considerably affected by various environmental conditions (Suárez and Marañón, 2003; Lee et al., 2009; Joo et al., 2014; Song et al., 2016; Yun et al., 2018). Some previous studies have suggested that nutrients may lead to different carbon allocation patterns in the photosynthetic end-products of phytoplankton in the region (Lee et al., 2009; Joo et al., 2014; Song et al., 2016). Indeed, Lee et al. (2009) observed largely different carbon allocation patterns under different water masses, which was mainly explained by the different nutrient distributions. In addition, they found that the vertical trends of carbon allocation could result from increasing major nutrient concentrations with depth (Lee et al., 2009). Song et al. (2016) also suggested that a high protein production rate of phytoplankton appears to be sustained by high concentrations of major nutrients in the Antarctic Ocean. However, distinct vertical patterns in photosynthetic carbon allocation into proteins were not observed in this study, even though the nutrient conditions at the 1% light depths were higher than those at the surface (Figure 5). Moreover, the phytoplankton sampled in this study were under a considerably nitrogen-deficient environment throughout the euphotic zone in the region, as can be deduced from the low N:P ratio and N:Si ratio (as discussed in the section above). Therefore, nutrients might not have been a major factor affecting carbon allocation into different macromolecules of phytoplankton in the northern Chukchi Sea during our cruise periods in 2011 and 2012.

In addition to nutrients, irradiance could be an important factor affecting differences in carbon allocation in the photosynthetic biochemical composition of phytoplankton (Smith and Morris, 1980; Smith et al., 1987; Cota and Smith, 1991; Suárez and Marañón, 2003). In the Antarctic Ocean, Smith and Morris (1980) found that phytoplankton incorporated as much as 80% of fixed carbon into lipids, whereas they showed less incorporation into proteins under conditions of low light intensity and low temperature. They explained that incorporation into lipids contributed approximately 20% of the fixed carbon and that the synthesis of polysaccharides and proteins was more prominent as temperatures (>0°C) and light intensities increased (Smith and Morris, 1980). Smith et al. (1989) also suggested that cold and low-light environments can cause high lipid synthesis rates in arctic ice algae. According to Larson and Rees (1996), increased chl a production with a corresponding nitrogen demand for the synthesis of the tetrapyrrole ring of chl a could occur in low-light acclimated algae. As a result, low carbon allocation into proteins could be anticipated due to increased pigment synthesis under low-light conditions (Mock and Gradinger, 2000). In the present study, the temperature between the 2 years was not significantly different (*p* > 0.05), as it was below 0°C (Table 3). However, the light conditions affecting phytoplankton in the water column could be considerably different because of sea ice conditions. Presumably, better abundant irradiance penetrating within the upper ocean could be expected under almost ice-free conditions in 2012 compared to 2011. As a result, higher incident irradiance under ice-free environments in 2012 could be more favorable to polysaccharide and protein production than lipid production. In contrast, low-light conditions under high sea ice concentrations in 2011 could have led to high lipid and low protein allocation in this study. Indeed, PCA revealed that the lipid fraction presented a close positive relationship with sea ice conditions (Figure 7A).

In fact, we compared the photosynthetic carbon allocation of phytoplankton according to sea ice conditions (Table 4). Indeed, the photosynthetic carbon allocation into different macromolecular pools was significantly different depending on the sea ice conditions. The carbon allocation into polysaccharides and proteins in ice-free and intermediate regions was significantly (*t*-test, *p* < 0.01 and *p* < 0.05, respectively) greater than that in the ice-heavy regions (Table 4). In contrast, the carbon allocation into lipids in ice-free and intermediate regions was significantly (*t*-test, *p* < 0.01) lower than that in ice-heavy regions. These different responses of phytoplankton to sea ice conditions can be explained by light availability because the interception of light by sea ice cover causes poor light conditions for phytoplankton in the water column under sea ice cover (Rysgaard et al., 1999; Sakshaug, 2004). According to Leu et al. (2006), the light also had a pronounced effect on the fatty acid composition of phytoplankton during the stratified periods in a high Arctic fjord. Therefore, the light condition could be an important factor causing different photosynthetic carbon allocation patterns of phytoplankton in this study. Notably, there was a strong negative correlation between the protein and lipid composition (*r* = −0.75, *p* < 0.01), implying that high relative rates of allocation to the protein fraction in 2012 could have occurred primarily at the expense of lipid synthesis (Figure 6).

**TABLE 4:** Photosynthetic carbon allocation in phytoplankton under different sea ice conditions.

	**Ice-free and intermediate regions**	**Ice-heavy regions**
LMWM (low-molecular-weight metabolites) (%)	11.0 (16.8)	26.0 (14.4)
Lipids (%)	24.3 (21.7)	41.5 (14.7)
Polysaccharides (%)	26.7 (20.2)	10.1 (8.5)
Proteins (%)	37.9 (22.5)	22.4 (15.0)

*The experimental stations were divided into two regions: ice-free and intermediate regions and ice-heavy regions, following Yun et al. (2012). Ice-free (<10% ice coverage) and intermediate regions (30–60% ice coverage) (*n* = 13) (a) and ice-heavy regions (>80% ice coverage) (*n* = 5) (b). Mean values are given (standard deviation in parentheses).*

### Influence of Major Taxonomic Groups on Carbon Allocation Patterns of Phytoplankton

In the western Arctic Ocean, flagellates are numerically dominant and are mainly supported by regenerated nutrients (Carmack et al., 2004; Tremblay et al., 2008; Brugel et al., 2009). In this study, we also observed that the community was generally dominated by nano- and pico-sized flagellates in both 2011 and 2012. However, the proportions of the total biovolume represented by the different phytoplankton groups were different between the 2 years (Figure 4). Flagellates contributed 48.6% of the total biovolume, and the contribution of diatoms was 31.1% in 2011, whereas diatoms and flagellates contributed 49.0 and 24.5% in 2012, respectively. Unidentified nano- and pico-sized phytoplankton contributed 20.4 and 26.5% of total biovolume in 2011 and 2012, respectively. In particular, the contributions of flagellates were significantly different between the 2 years (*p* < 0.05) (Table 3). These different patterns in terms of phytoplankton size classes might affect the different patterns of phytoplankton carbon allocation in this study. According to Li and Platt (1982), species composition may be important in determining pathways of carbon assimilation. Indeed, a previous study reported that the relative rates of lipid synthesis could be associated with species composition (Fernandez et al., 1994). For example, carbon allocation into lipids is greater under dominance of flagellates, whereas lower lipid synthesis is observed in association with dominance of diatoms (see the Fernandez et al., 1994). When we applied this observation to our study, the predominant lipid and protein fractions in 2011 and 2012 coincided with the dominance of flagellates and diatoms, respectively. Indeed, PCA revealed significantly close relationships between flagellates and the lipid fraction and diatoms and the protein proportion (Figure 7B). Additionally, we compared the specific lipid production rates between the 2 years. The specific lipid production rates in 2011 and 2012 were 0.00035 and 0.00005 h^–1^, respectively. The lipid production rate under dominance of flagellates in 2011 was significantly higher than that under diatom dominance in 2012 (*t*-test, *p* < 0.001), even though the specific protein production rate was not considerably different between 2011 and 2012 (*t*-test, *p* > 0.05). Therefore, the different phytoplankton groups based on size classes might lead to changes in the pattern of carbon allocation into each biochemical pool, especially for the lipid fraction.

In conclusion, this study reported the different patterns of photosynthetic carbon allocation in phytoplankton in the northern Chukchi Sea under different sea ice conditions. More photosynthetic carbon allocation to lipids and considerably less to proteins was found among photosynthetic end-products under heavy sea ice conditions, whereas a high-protein and low-lipid composition was observed under ice-free and intermediate sea ice conditions. These different carbon allocation patterns responding to the sea ice conditions may be related to light availability, since less carbon allocation into proteins could occur due to higher pigment synthesis under low-light conditions (Mock and Gradinger, 2000). In addition, the differences in major phytoplankton groups that can be inferred by biovolume seem to affect the composition of the photosynthetic end-products of phytoplankton, since a high lipid production rate was closely linked to the dominance of flagellates. Given the results of this study, we conclude that the physiological status of phytoplankton is currently changing under altered sea ice conditions and that it would be further changed under ongoing environmental changes in the Arctic Ocean.

At present, it is obvious that the primary production of phytoplankton is increasing under the increased light penetration to the upper ocean and lengthened melt season, which are mainly caused by sea ice changes in the Arctic Ocean (Arrigo and van Dijken, 2011, 2015; Stroeve et al., 2012, 2014; Kahru et al., 2016). Accordingly, our knowledge of the changes in physiological status of Arctic phytoplankton should be extended. Obviously, the quantitative (i.e., biomass or productivity) and qualitative (i.e., biochemical composition or physiological aspect) changes in the phytoplankton community driven by climate change could impact higher trophic levels. According to previous studies, changes in the quantity and quality of the prey (i.e., phytoplankton) of zooplankton could affect the feeding, development, reproduction periods and survival of zooplankton (Cook et al., 2007; Søreide et al., 2010; Daase et al., 2011). Indeed, the biogeographic distribution of copepod species of the genus *Calanus*, as fundamental components of the Arctic ecosystem, is closely related to changes in phytoplankton as well as physical environments such as sea ice or upper ocean temperatures (Feng et al., 2017).

Regarding the changes in photosynthetic end-products, the dominant protein composition under reduced sea ice conditions might result in low caloric contents of phytoplankton as an energetic perspective and subsequently lead to changes in the nutritional and energetic strategies of zooplankton, even though the biochemical composition of phytoplankton cannot be directly linked to predators (Yun et al., 2015b; Jo et al., 2017; Kang et al., 2017). In particular, noticeable recent changes in the phytoplankton size structure, such as pico-sized phytoplankton-based ecosystems (Li et al., 2009) or the increase of temperate phytoplankton under the warming condition of the Arctic (Neukermans et al., 2018), could be important in leading the shift to the different photosynthetic end-products of phytoplankton in a rapidly changing Arctic Ocean. More studies are still needed to better understand how phytoplankton physiological status responds to ongoing and future changes in Arctic environments and in turn impacts local and regional marine ecosystems through the food web.

## Data Availability Statement

All datasets generated for this study are included in the manuscript/Supplementary Files.

## Author Contributions

SL conceived the ideas and designed methodology. MY wrote the manuscript and conducted lab experiment and data analysis. HJ, JK, and JP performed the field experiments. JL conducted the statistical analysis. S-HK was the leader of the Korean Arctic Research Program and provided scientific advice. JS critically reviewed the manuscript. All authors approved the final version of the manuscript.

## Conflict of Interest

The authors declare that the research was conducted in the absence of any commercial or financial relationships that could be construed as a potential conflict of interest.

## Supplementary Material

TABLE S1 Phytoplankton species (cell abundance, cells L^–1^) at three optical depths (100, 30, and 1% optical depths) at stations where photosynthetic end-product measurements were conducted in 2011.

TABLE S2 Phytoplankton species (cell abundance, cells L^–1^) at three optical depths (100, 30, and 1% optical depths) at stations where photosynthetic end-product measurements were conducted in 2012. Samples were not obtained at the 100% optical depth at station 26.
