# Supplementary material for: Potential Implications of Changing Photosynthetic End-Products of Phytoplankton Caused by Sea Ice Conditions in the Northern Chukchi Sea
Source: Front Microbiol. 2019 Oct 2;10:2274. doi: 10.3389/fmicb.2019.02274 (PMC6783801; doi:10.3389/fmicb.2019.02274)
Supplement: TABLE S1 — Phytoplankton species (cell abundance, cells L–1) at three optical depths (100, 30, and 1% optical depths) at stations where photosynthetic end-product measurements were conducted in 2011. [file Data_Sheet_1.PDF]

Table S1

	1			2			3			4			7			9			11			13			15			17		
	100%	30%	1%	100%	30%	1%	100%	30%	1%	100%	30%	1%	100%	30%	1%	100%	30%	1%	100%	30%	1%	100%	30%	1%	100%	30%	1%	100%	30%	1%
**Dinophyceae**																														
Dinophysis acuta																			1756											
Dinophysis sp.																				1756										
Gonyaulax sp.																														
Gonyaulax cyst form										3512	1756			3512																
Gymnodinium sp.				7023	1756					5267																				
Gyrodinium sp.	1756												1756			3512						1756	3512		1756	1756			1756	
Prorocentrum sp.				3512																			3512							
Dinoflagellate cyst form	28971	10535	878	1756	3512																									
**Bacillariophyceae**																														
Actinocyclus sp.																				1756										
Chaetoceros compressus	14047	5267	14925								7023																			
Chaetoceros concavicornis		1756																												
Chaetoceros curvicetus	5267	3512	10535				12291																							
Chaetoceros danicus																			1756											
Chaetoceros debilis	19314	9657	17558																											
Chaetoceros decipiens						7023					1756																			
Chaetoceros sp.	10535	7023	230014		5267	3512	1756	3512			3512								3512											
Chaetoceros cyst form						18030178		43896																						
Fragilariopsis sp. (> 20㎛)	8779																									10535				
Melosira sp.																		5267												
Minidiscuss sp.																												1756		
Navicula sp.	2634	2634	19314			1756	1756									1756		1756					3512				12291			
Nitzschia sp.	2634																							1756						
Pseudonitschia sp.	76818	131687				5267								43896																
Thalassionema sp.	2634	7023				7023																								
Thalassiosira eccentrica			17558																											
Thalassiosira nordenskioldii			44774			3512																								
Thalassiosira sp. (>20㎛, <200㎛)			46529			5267						3512	3512										1756							
**Cryptophyceae**																														
Cryptomonas sp.																	10974									87791				
**Chrysophyceae**																														
Dinobryon belgica																						616296								
**Dictyochophyceae**																														
Dictyocha speculum														1756	1756						5267						10535			5267
Meringosphaera mediterranea	1756																10535			5267		5267	1756		3512		3512	1756		
**Prasinophyceae**																														
Pyramimonas sp.	98765	76818		10974																		131687	32922		460905	164609				
**Prymneosiophyceae**																														
Phaeocystis sp.																								76818			21948		10974	32922
unidentified sp. (nano size)	230453	164609	340192	515775	351166	131687	131687	186557	131687	669410	54870	120713	65844	164609		285322	274348	10974	373114	230453	87791	351166	395062	65844	307270	548697	131687	603567	175583	142661
unidentified sp. (pico size)	504801	329218	1668038	790123	866941	43896	329218	109739	164609	866941	998628	230453	592593	373114	208505	570645	427984	153635	581619	285322	373114	680384	504801	142661	592593	1218107	109739	1294925	910837	186557
**Total abundance (cells L^{-1})**	1.01E+06	7.50E+05	2.41E+06	1.33E+06	1.23E+06	1.82E+07	4.77E+05	3.44E+05	2.96E+05	1.55E+06	1.07E+06	3.55E+05	6.64E+05	5.87E+05	2.10E+05	8.61E+05	7.24E+05	1.72E+05	9.62E+05	5.25E+05	4.66E+05	1.79E+06	9.47E+05	2.87E+05	1.37E+06	2.03E+06	2.90E+05	1.90E+06	1.10E+06	3.67E+05
**Number of species**	13	10	9	4	3	8	3	2	N. D	2	4	1	2	3	N. D	2	2	2	3	3	1	4	6	2	3	4	4	2	2	2
**Diversity index**	1.53	1.53	1.07	0.77	0.65	0.07	0.75	1.01	0.69	0.72	0.29	0.69	0.37	0.89	0.05	0.67	0.80	0.43	0.72	0.78	0.54	1.27	0.90	1.07	1.09	1.03	1.23	0.64	0.51	0.99

Table S2

	5			13			19			26			30			36			40			42		
	100%	30%	1%	100%	30%	1%	100%	30%	1%	100%	30%	1%	100%	30%	1%	100%	30%	1%	100%	30%	1%	100%	30%	1%
**Dinophyceae**																								
Alexandrium sp.											20													
Ceratium arcticum					10		10																	
Gyrodinium sp. 1					3512		878				7023									12291				
Dinoflagellate cyst form			10535																				5267	
**Bacillariophyceae**																								
Actinocyclus sp.							2634																	
Asteromphalus sp.												70												
Chaetoceros affinis			1756				4390																	
Chaetoceros atlanticus																7023								
Chaetoceros compressus				6145	7023		14925																	
Chaetoceros concavicornis																								
Chaetoceros convolutus																								
Chaetoceros curvicetus																								
Chaetoceros danicus							2634									10535								
Chaetoceros debilis							10535									6145								
Chaetoceros decipiens							5267									5267								
Chaetoceros diadema																								
Chaetoceros didymus																								
Chaetoceros furcellatus																								
Chaetoceros mitra							1756																	
Chaetoceros peruvianus																								
Chaetoceros simplex																								
Chaetoceros socialis						10535																		
Chaetoceros sp.			28093				15802		1756			3145862				6145								
Coconeis sp.							1756															878		
Cosinodiscus sp.									20															
Cylindrotheca sp.					3512						21948	8779						18290						
Fragilariopsis sp. (> 20㎛)							1756					158025	878				5267							
Fragilariopsis sp. (< 20㎛)		1756	32922			36580		14047	21948		93059								10974		10974		12291	
Fragilaria sp.							27215																	
Melosira sp.												5853										12291	22826	
Minidiscuss sp.	10974	21948			21948		10974										1756		21948			54870	18290	
Navicula sp.			1756				2634				12291	658436		10								5267	3512	
Nitzschia sp.						36580																7023		
Pleurosigma sp.					1756																			
Pseudonitschia sp.												695016												
Rhizosolenia hebatata								90																
Rhizosolenia sp.							80																	
Thalassionema nitzschioides			19314				9657	17558														8779		
Thalassionema sp.						3512		3512			1756	35117				1756					7023			
Thalassiosira bulbosa												43896												
Thalassiosira eccentrica				10			2634	5267			10535	169730												
Thalassiosira hyalina												55601												
Thalassiosira nordenskioldii												67307												
Thalassiosira sp. (>20㎛, <200㎛)			10535	878			9657	7023	1756			125834					1756				3512		3512	
Thalassiosira sp. (<20㎛)				10974		1756												1756						
**Cryptophyceae**																								
Cryptomonas sp.							153635		43896													32922	91449	
**Chrysophyceae**																								
Dinobryon belgica							126420	54431			217723					34239						5267		
**Dictyochophyceae**																								
Dictyocha speculum			10535			14047	878		3512						3512		1756	3512		1756	5267	4390	1756	
Meringosphaera mediterranea			3512				878	10535					878				3512				3512			
**Prasinophyceae**																								
Pyramimonas sp.			526749		43896			21948														54870	54870	
**Prymneosiophyceae**																								
Phaeocystis sp.					32922	310928	21948	21948																54870
unidentified sp. (nano size)	296296	153635	219479	120713	175583	219479	1218107	285322	153635		87791	128029	197531	54870	131687	351166	120713	237769	43896	65844	142661	164609	219479	153635
unidentified sp. (pico size)	482853	208505	417010	318244	801097	3932327	340192	548697	855967		1722908	91449	351166	406036	427984	164609	581619	1883859	285322	274348	746228	351166	1993599	614540
Total abundance (cells L^{-1})	7.90E+05	3.86E+05	1.28E+06	4.57E+05	1.09E+06	4.57E+06	1.99E+06	9.90E+05	1.08E+06	N.D	2.18E+06	5.39E+06	5.50E+05	4.61E+05	5.63E+05	5.87E+05	7.16E+05	2.15E+06	3.62E+05	3.54E+05	9.19E+05	7.02E+05	2.43E+06	8.23E+05
Number of species	1	2	10	4	8	97	24	10	6	N.D	8	13	2	1	1	7	5	3	2	2	5	10	9	1
Diversity index	0.73	0.89	1.43	0.76	0.91	0.58	1.36	1.28	0.71	N.D	0.81	1.49	0.67	0.37	0.58	1.11	0.58	0.42	0.72	0.65	0.62	1.51	0.73	0.71
